# The Progestin Receptor Interactome in the Female Mouse Hypothalamus: Interactions with Synaptic Proteins Are Isoform Specific and Ligand Dependent

**DOI:** 10.1523/ENEURO.0272-17.2017

**Published:** 2017-09-20

**Authors:** Kalpana D. Acharya, Sabin A. Nettles, Katherine J. Sellers, Dana D. Im, Moriah Harling, Cassandra Pattanayak, Didem Vardar-Ulu, Cheryl F. Lichti, Shixia Huang, Dean P. Edwards, Deepak P. Srivastava, Larry Denner, Marc J. Tetel

**Affiliations:** 1 Neuroscience Program, Wellesley College, Wellesley, MA 02481, USA; 2 Department of Basic and Clinical Neuroscience, Maurice Wohl Clinical Neurosciences Institute, Institute of Psychiatry, Psychology and Neuroscience, and MRC Centre for Neurodevelopmental Disorders, King’s College London, London, UK; 3 Quantitative Analysis Institute, Departments of Mathematics and Quantitative Reasoning, Wellesley College, Wellesley, MA 02481, USA; 4 Chemistry Department, Boston University, Boston, MA 02215, USA; 5 Department of Pathology and Immunology, Washington University School of Medicine, St. Louis, MO 63110; 6 Department of Molecular and Cellular Biology, Department of Pathology and Immunology, and Dan L. Duncan Cancer Center, Baylor College of Medicine, Houston, TX 77030, USA; 7 Department of Internal Medicine, University of Texas Medical Branch, Galveston, TX 77555, USA

**Keywords:** Cortex, estrogen, progesterone, proteomics, synapse, synapsin

## Abstract

Progestins bind to the progestin receptor (PR) isoforms, PR-A and PR-B, in brain to influence development, female reproduction, anxiety, and stress. Hormone-activated PRs associate with multiple proteins to form functional complexes. In the present study, proteins from female mouse hypothalamus that associate with PR were isolated using affinity pull-down assays with glutathione S-transferase–tagged mouse PR-A and PR-B. Using complementary proteomics approaches, reverse phase protein array (RPPA) and mass spectrometry, we identified hypothalamic proteins that interact with PR in a ligand-dependent and isoform-specific manner and were confirmed by Western blot. Synaptic proteins, including synapsin-I and synapsin-II, interacted with agonist-bound PR isoforms, suggesting that both isoforms function in synaptic plasticity. In further support, synaptogyrin-III and synapsin-III associated with PR-A and PR-B, respectively. PR also interacted with kinases, including c-Src, mTOR, and MAPK1, confirming phosphorylation as an integral process in rapid effects of PR in the brain. Consistent with a role in transcriptional regulation, PR associated with transcription factors and coactivators in a ligand-specific and isoform-dependent manner. Interestingly, both PR isoforms associated with a key regulator of energy homeostasis, FoxO1, suggesting a novel role for PR in energy metabolism. Because many identified proteins in this PR interactome are synaptic proteins, we tested the hypothesis that progestins function in synaptic plasticity. Indeed, progesterone enhanced synaptic density, by increasing synapsin-I–positive synapses, in rat primary cortical neuronal cultures. This novel combination of RPPA and mass spectrometry allowed identification of PR action in synaptic remodeling and energy homeostasis and reveals unique roles for progestins in brain function and disease.

## Significance Statement

Progesterone binds to the progestin receptor (PR) isoforms, PR-A and PR-B, in the brain to profoundly influence female reproduction, brain development, neuroprotection, and stress. We combined mass spectrometry and reverse phase protein arrays to identify mouse hypothalamic proteins that function in synaptic plasticity, transcription, translation, and energy metabolism and interacted with PR in a ligand-dependent and isoform-specific manner. In addition, progesterone increased synaptic density in primary cortical neurons, suggesting a function for progesterone in synapse formation in cortical neurons. Taken together, these findings provide evidence for novel functions of PR in synaptic modulation and energy homeostasis to affect health and disease.

## Introduction

Progestins, a class of steroid hormones, act in the brain to profoundly influence development (Beyer et al., 2002; Wagner, 2006); neuroprotection (Stein and Hoffman, 2003; Singh, 2006; Irwin et al., 2008; Su et al., 2012); reproductive behavior (Sinchak and Micevych, 2001; Blaustein, 2008); learning, memory, and cognition (Hull et al., 1980; Sandstrom and Williams, 2001; Wagner, 2006); and anxiety and stress (Mora et al., 1996; Blaustein and Ismail, 2013; Keyes et al., 2013; Uphouse et al., 2013). In addition, progestins have been implicated in a variety of human diseases, including neurodegeneration and breast cancer (Richer et al., 2002; Brinton et al., 2008; Obr and Edwards, 2012; Diep et al., 2015).

Progestins elicit many of their effects by binding to progestin receptors (PR), which exist as two isoforms in mammals, an N-terminally truncated PR-A and a full-length PR-B (Tetel and Lange, 2009). Both isoforms share a C-terminal ligand binding domain, a DNA binding domain, and two transactivation domains (Horwitz et al., 1990; Kastner et al., 1990). Interestingly, a third transactivation domain exclusive to PR-B, and a PR-A specific inhibitory region, have been identified, allowing differential functions by the isoforms (Hovland et al., 1998). Isoform-specific transcriptional activation by PR has been observed *in vitro* and *in vivo* (Sartorius et al., 1994; Giangrande et al., 1997; Conneely et al., 2000; Cheng et al., 2001; Richer et al., 2002; Mulac-Jericevic et al., 2003). Isoform-specific knockout mice reveal that although PR-A in the mouse hypothalamus is the primary mediator of female sexual behavior, both isoforms are required for the full behavioral response (Mani et al., 2006). In support, PR-A and PR-B are differentially expressed in the female mouse hypothalamus (Acharya et al., 2015).

PR associate with multiple proteins, including nuclear receptor coactivators, which stabilize the receptor complex and enhance transcription (Hill et al., 2012; Goswami et al., 2014; Simons et al., 2014). In the female mouse hypothalamus, the PR isoforms are differentially expressed with steroid receptor coactivator-1 (SRC-1, also named NCOA1) and SRC-2 (NCOA2; Acharya et al., 2015). In addition, SRC-1 and SRC-2 from hypothalamus associate with the PR isoforms in a ligand-dependent and isoform-specific manner (Molenda-Figueira et al., 2008; Yore et al., 2010) and modulate hormone-dependent gene expression and female sexual behavior (Apostolakis et al., 2002; Molenda et al., 2002; Molenda-Figueira et al., 2006). Taken together, these studies indicate that nuclear receptor coactivators are important in PR function in the brain.

PR act through both classic and nonclassic pathways in the brain and other progestin-responsive tissues. For example, in the hypothalamus, PR activation induces lordosis in female rodents through transcriptional regulation (Leonhardt et al., 2003; Molenda-Figueira et al., 2006) as well as nonclassic kinase activation (González-Flores et al., 2004; Mani et al., 2006). PRs are present in synapses (Waters et al., 2008; Mitterling et al., 2010) and rapidly alter dendritic spine densities in rat hippocampus (Woolley and McEwen, 1993; McEwen and Woolley, 1994), cortical neurons (Chen et al., 2009; Sanchez et al., 2013), and hypothalamus (Griffin et al., 2010).

Although we are gaining a better understanding of distinct functions of the PR isoforms in brain, little is known about the mechanisms involved. Therefore, to investigate the factors that could contribute to the differential function of the PR isoforms in brain, we have combined mass spectrometry (MS) and reverse phase protein array (RPPA) in a functional proteomic analysis to identify proteins from adult female mouse hypothalamus that interact with mouse PR-A and PR-B. In addition, given the identification of many synaptic proteins that interact with PR in the present study, we tested the hypothesis that progestins influence synaptic plasticity by increasing synapse formation in cortical neurons.

## Materials and Methods

### Animals

Female C57/BL6 mice were bred in the Wellesley College Animal Facility (Wellesley, MA). Mice were group-housed (three to six/group) under a 12-h light/dark cycle. Food and water were available *ad libitum*. All animal procedures were approved by the Institutional Animal Care and Use Committee of Wellesley College and were conducted in accordance with the National Institutes of Health Guide for the Care and Use of Laboratory Animals.

### Tissue collection and preparation

Mice (8 to 10 wks old) were bilaterally ovariectomized under 1.5% isoflurane. One week later, mice were killed by asphyxiation under CO_2_. Brains were quickly removed, and hypothalamic tissue was dissected using coordinates from the mouse brain atlas (Paxinos and Franklin, 2004), immediately frozen on dry ice, and stored at –80°C until homogenization. Hypothalami from four to eight mice were pooled to generate each sample and lysed in lysis buffer containing 10 mm Tris, 10% glycerol, 400 mm NaCl, 1 mm dithiothreitol (DTT), and 1 mm EDTA (pH 7.4) with protease inhibitors (1:10 dilution, P2714, Sigma-Aldrich). Lysates were incubated on ice for 30 min followed by centrifugation at 13,200 × *g* for 30 min at 4°C. Supernatants containing proteins were stored at –80°C.

### Glutathione S-transferase–tagged mouse progestin receptors

Mouse PR-B cDNA was cloned into the CMV-based mammalian cell expression plasmid pcDNAI (Invitrogen) by insertion into the PspHI/EcoRI site. Mouse PR-A cDNA was prepared by partial digest of mouse PR-B cDNA, ligated into the pBlueBacHis2B transfer plasmid, and inserted into the BamHI/EcoRV site of pcDNAI. pAcG2T baculovirus expression vector (BD Biosciences) was used for expression of PR-A and PR-B as fusion proteins, containing an N-terminal glutathione S-transferase (GST) tag. Site-directed mutations were performed using QuikChange XL Site-directed Mutagenesis Kit (Stratagene). The mutated sites were used to ligate cDNAs of mouse PR-A and PR-B into the respective restriction sites of pAcG2T. Mouse PR-A containing BamHI and EcoRI sites was ligated into the respective sites of the mutated pAcG2T. Similarly, mouse PR-B containing EcoRI and NotI sites was ligated into the respective sites of the mutated pAcG2T. Both DNA constructs were fully sequenced from the start of the GST to the end of the mouse PR subtype (Genewiz).

After the full sequences were confirmed, recombinant proteins containing full-length mouse PR-A or PR-B fused to a GST tag were expressed in *Spodoptera frugiperda* (Sf9) insect cells by the Baculovirus/Monoclonal Antibody Facility of the Baylor College of Medicine as described previously (Tetel et al., 1999; Molenda-Figueira et al., 2008; Yore et al., 2010). Insect cell cultures for GST-PR were incubated with saturating doses of 200 nm of the PR agonist R5020 or in the absence of PR ligand. Sf9 cell pellets were homogenized in buffer containing 10 mm Tris, 10% glycerol, 400 mm NaCl, 1 mM DTT, and 1 mm EDTA (pH 7.4) with protease inhibitors. Homogenates were incubated on ice for 30 min, and then centrifuged at 13,200 × *g* for 30 min at 4°C. Supernatants containing proteins were stored at –80°C.

### GST-PR pull-down

GST-PR pull-down assays were performed as described previously (Molenda-Figueira et al., 2008; Yore et al., 2010). Briefly, glutathione Sepharose 4B packed resin (50 µL, 0.05 mg/mL, GE Healthcare) was added to siliconized centrifuge tubes and pretreated with ovalbumin (1 mg/mL, Thermo Fisher Scientific) for 15 min on an end-over-end rotator at 4°C. Resin was rinsed three times with TG buffer (20 mm Tris–HCl and 10% glycerol) containing 1 m urea and 100 mm NaCl (TG-NaCl-U, pH 8.0). GST-PRA or GST-PRB bound to the agonist R5020, or unliganded, were used for pull-downs. R5020 is a synthetic progestin that binds with a similar affinity to both PR-A and PR-B (Skafar, 1991; Carbajo et al., 1996). Equal amounts of recombinant mouse GST-PR suspended in TG buffer with 1 m urea (TG-U, pH 8.0) were added to resin and incubated on a rotator at 4°C for 1 h. After incubation, resin with immobilized GST-PR was washed four times with TG-NaCl-U (with reducing urea concentrations of 1, 0.5, 0.25, and 0 m). Equal amounts of pooled hypothalamic homogenates were added to immobilized GST-PR and incubated on a rotator for 1 h at 4°C. The resins were washed four times with TG-NaCl (without urea).

Proteins bound to GST-PR resin were eluted in specific buffers for each of the analyses. For Western blots, samples were eluted by boiling for 5 min in 2× Laemmli sample buffer (Bio-Rad) with 35 mm DTT (Sigma-Aldrich) for 5 min. For MS analysis, GST-PR and proteins bound to resin were eluted by incubating in 250 mm glutathione elution buffer (in 50 mm Tris-HCl, pH 8.0) at 4°C for 10 min. Samples for RPPA analysis were boiled in SDS sample buffer (Invitrogen) with 2.5% β-mercaptoethanol for 5 min.

### Mass spectrometry analysis

Pull-down assays for MS were performed with GST-PRA or GST-PRB, unliganded or bound to R5020. Tryptic peptides prepared as previously described (Molenda-Figueira et al., 2008) for each receptor type and ligand condition were block-randomized and analyzed in triplicate (Oberg and Vitek, 2009) by nano-LC-MS/MS using a nano-LC chromatography system (UltiMate 3000 RSLCnano, Dionex), coupled online to a Thermo Orbitrap Fusion mass spectrometer (Thermo Fisher Scientific) through a nanospray ion source (Thermo Fisher Scientific). Chromatographic columns were made from 75-µm-ID polyimide-coated fused silica capillary (Polymicro Technologies) packed with 5 µm Zorbax SB-C18 reversed-phase packing (Agilent) to a length of 10 cm by using a Pressure Injection Cell (NextAdvance). The trap column was prepared in the same manner but to a length of 1 cm. After equilibrating the column in 98% solvent A (0.1% formic acid in water) and 2% solvent B (0.1% formic acid in acetonitrile), the samples (3 µL in solvent A) were injected onto the trap column and eluted (400 nL/min) by gradient elution onto the C18 column as follows: isocratic at 2% B, 0–5 min; 2% to 32% B, 5–49 min; 32% to 90% B, 49–50 min; isocratic at 90% B, 50–54.5 min; 90% to 2% B, 54.5–55 min; and isocratic at 2% B, 55–60 min. All LC-MS/MS data were acquired using XCalibur, version 2.1.0 (Thermo Fisher Scientific) in positive ion mode using a top speed data-dependent acquisition method with a 2-s cycle time. The survey scans (*m*/*z* 350–1600) were acquired in the Orbitrap at 120,000 resolution (at *m*/*z* 400) in profile mode, with a maximum injection time of 50 ms and an automatic gain control (AGC) target of 400,000 ions. The S-lens RF level was set to 60. Isolation was performed in the quadrupole with a 1.6-Da isolation window, and CID MS/MS acquisition was performed in profile mode in the ion trap using rapid scan rate, with the following settings: parent threshold, 10,000; isolation width, 1.6 Da; normalized collision energy, 35%; maximum injection time, 150 ms; and AGC target, 10,000 ions. Monoisotopic precursor selection and charge state filtering were on, with charge states 2–4 included. Dynamic exclusion was used to remove selected precursor ions for 60 s after acquisition of one MS/MS spectrum.

### Proteomic data analysis of MS samples

All data files were searched against a Uniprot mouse database (June 2014 version, 16,728 proteins), appended with the Common Repository of Adventitious Proteins contaminant database using PEAKS (Ma et al., 2003; Han et al., 2004, 2011; Zhang et al., 2012). Searches were performed with a parent ion tolerance of 10 ppm, fragment ion tolerance of 0.80 Da, fixed modification of carbamidomethyl (C), and variable modifications of oxidation (M) and phosphorylation (STY). Trypsin was specified as the enzyme, allowing for two missed cleavages and one nonspecific cleavage. False discovery rate (FDR) estimation was allowed. The resulting peptide-spectrum matches >1% FDR were used to generate a spectral library for subsequent quantitative analysis. Data files for each pair of samples were imported into Skyline (MacLean et al., 2010) for label-free quantitative analysis (Schilling et al., 2012). After manual verification of all peaks, triplicate peptide intensities were used to calculate fold changes and *p*-values.

### Reverse phase protein array analysis

Hypothalamic tissue from adult female mice (*n* = 32) was pooled into four different groups. Similar to MS, RPPA assays were performed using pull-downs with GST-PRA and GST-PRB bound to R5020 or unliganded. RPPA assays were conducted as described previously (Chang et al., 2015) with the following modifications. Proteins associating with GST-PR bound to resins were eluted in 2× Tris-glycine SDS buffer with 2.5% β-mercaptoethanol and mixed with modified Tissue Protein Extraction Reagent (Pierce and Xu, 2010) and a cocktail of protease and phosphatase inhibitors (Roche Life Science; Chang et al., 2015). A 2470 Arrayer (Aushon BioSystems) with a 40-pin (185 µm) configuration was used to spot samples and control lysates onto nitrocellulose-coated slides (Grace Bio-labs) using an array format of 960 lysates/slide (2880 spots/slide). The slides were processed as described previously (Chang et al., 2015) and probed with a set of 212 antibodies (Table 1) against 140 total proteins and 72 proteins phosphorylated on specific sites using an automated slide stainer, Autolink 48 (Dako). Each slide was incubated with one specific primary antibody. Negative control slides were incubated with antibody diluent instead of primary antibody. Primary antibody binding was detected using a biotinylated secondary antibody followed by streptavidin-conjugated IRDye680 fluorophore (LI-COR Biosciences). The total protein content of each spotted lysate was assessed by fluorescent staining with Sypro Ruby Protein Blot Stain according to the manufacturer’s instructions (Invitrogen).

**Table 1.** List of antibodies used for RPPA analysis

**Antibody name**	Company	Catalog #	Host
14-3-3zeta,gamma,eta_R_V	EMD Millipore (Upstate)	06-408	Rabbit
Akt_R_V	CST	9272	Rabbit
*p*-Akt(S473)_R_V	CST	9271	Rabbit
*p*-Akt(T308)_R_V	CST	9275	Rabbit
*p*-ALK(3B4)(Y1586)_R_V	CST	3348	Rabbit
*p*-ALK(Y1604)_R_V	CST	3341	Rabbit
*p*-AMPKa(40H9)(T172)_R_V	CST	2535	Rabbit
*p*-AMPKa1(S485)_R_V	CST	4184	Rabbit
*p*-AMPKb1(S108)_R_V	CST	4181	Rabbit
Annexin1_R_V	Invitrogen	71-3400	Rabbit
ATM_R_V	Abcam	ab32420	Rabbit
ATR_R_V	CST	2790	Rabbit
*p*-ATR(S428)_R_V	CST	2853	Rabbit
*p*-AuroraA(T288)/B(T232)/C(T198)_R_V	CST	2914	Rabbit
AuroraA/AIK_R_V	CST	3092	Rabbit
*p*-Axl(D12B2)(Y702)_R_V	CST	5724	Rabbit
Bad_R_V	CST	9292	Rabbit
*p*-Bad(S112)_R_V	CST	9291	Rabbit
*p*-Bad(S136)_R_V	CST	9295	Rabbit
*p*-Bad(S155)_R_V	CST	9297	Rabbit
Bak_R_V	CST	3814	Rabbit
Bax_R_V	CST	2772	Rabbit
*p*-Bcl2(5H2)(S70)_R_V	CST	2827	Rabbit
*p*-Bcl2(T56)_R_V	CST	2875	Rabbit
BRCA1_R_V	EMD Millipore (Upstate)	07-434	Rabbit
BRCA2_R_V	CST	9012	Rabbit
Caspase-3_R_V	CST	9662	Rabbit
*p*-Beta-Catenin(S33/37/T41)_R_V	CST	9561	Rabbit
Caveolin-1(D46G3)XP_R_V	CST	3267	Rabbit
*p*-Caveolin-1(EPR2288Y)(Y14)_R_V	Abcam-Epitomics	2267-1	Rabbit
CD24(FL-80)_R_V	Santa Cruz Biotechnology	sc-11406	Rabbit
*p*-Chk1(S345)_R_V	CST	2341	Rabbit
*p*-Chk2(S33/35)_R_V	CST	2665	Rabbit
Claudin-1_R_V	CST	4933	Rabbit
c-Met_R_V	Abcam	ab51067	Rabbit
*p*-c-Myc(T58)_R_V	Abcam	ab28842	Rabbit
DKK1_R_V	CST	4687	Rabbit
EGFR(D38B1)XP_R_V	CST	4267	Rabbit
EGFR(L858R)_R_V	CST	3197	Rabbit
*p*-EGFR(S1046/1047)_R_V	CST	2238	Rabbit
*p*-EGFR(Y1045)_R_V	CST	2237	Rabbit
*p*-EGFR(Y1148)_R_V	CST	4404	Rabbit
*p*-EGFR(Y1173)_R_V	Invitrogen (BioSource)	44-794G	Rabbit
*p*-EGFR(Y1173)(53A5)_R_V	CST	4407	Rabbit
*p*-EGFR(Y1068)(D7A5)XP_R_V	CST	3777	Rabbit
Ezh2(D2C9)XP_R_V	CST	5246	Rabbit
FGFR1(D8E4)XP_R_V	CST	9740	Rabbit
FoxO1(C29H4)_R_V	CST	2880	Rabbit
FSP1/S100A4_R_V	EMD Millipore	Jul-74	Rabbit
*p*-HER2/ErbB2(Y1248)_R_V	CST	2247	Rabbit
*p*-HER2/ErbB2(Y877)_R_V	Imgenex	IMG-90185	Rabbit
HER3/ErbB3_R_V	CST	4754	Rabbit
*p*-HER3/ErbB3(Y1197)_R_V	CST	4561	Rabbit
HDAC1_R_V	CST	2062	Rabbit
HDAC3_R_V	CST	2632	Rabbit
HDAC4_R_V	CST	2072	Rabbit
HDAC6_R_V	Santa Cruz Biotechnology	sc-11420	Rabbit
ILK1_R_V	CST	3862	Rabbit
*p*-Jak1(Y1022/1023)_R_V	CST	3331	Rabbit
Kit-c_R_V	Abcam	ab32363	Rabbit
Lipocalin-1_R_V	Santa Cruz Biotechnology	sc-66943	Rabbit
LRP6_R_V	CST	2560	Rabbit
MEK1_R_V	Abcam-Epitomics	1235-1	Rabbit
*p*-MEK1/2(S217/221)_R_V	CST	9121	Rabbit
*p*-Met(Y1234/1235)_R_V	CST	3077	Rabbit
MMP-9_R_V	CST	3852	Rabbit
mTOR_R_V	CST	2972	Rabbit
*p*-mTOR(D9C2)XP(S2448)_R_V	CST	5536	Rabbit
Notch1(C44H11)_R_V	CST	3268	Rabbit
*p*-p27(T187)_R_V	Invitrogen (Zymed)	71-7700	Rabbit
*p*-p27/KIP1(T198)_R_V	Abcam	ab64949	Rabbit
p27/KIP1(C-term)_R_V	Abcam-Epitomics	ab32034(1591-1)	Rabbit
p38/MAPK_R_V	CST	9212	Rabbit
*p*-p38(D3F9) XP(T180/Y182)_R_V	CST	4511	Rabbit
p44/42MAPK(Erk1/2)_R_V	CST	9102	Rabbit
*p*-p44/42MAPK(Erk1/2)(T202/Y204)(197G2)_R_V	CST	4377	Rabbit
p53_R_V	CST	9282	Rabbit
*p*-p53(S15)_R_V	CST	9284	Rabbit
p70S6K_R_V	CST	9202	Rabbit
*p*-p70S6K(S371)_R_V	CST	9208	Rabbit
*p*-p70S6K(T389)_R_V	CST	9205	Rabbit
*p*-p70S6K(T412)_R_V	EMD Millipore (Upstate)	07-018	Rabbit
*p*-PDGFRa(23B2)(Y754)_R_V	CST	2992	Rabbit
PDGFRb(28E1)_R_V	CST	3169	Rabbit
*p*-PDK1(S241)_R_V	CST	3061	Rabbit
PIAS1_R_V	CST	3550	Rabbit
PTEN(D4.3)XP_R_V	CST	9188	Rabbit
*p*-PTEN(S380)_R_V	CST	9551	Rabbit
*p*-RafB(S445)_R_V	CST	2696	Rabbit
RANKL_R_V	Novus	NBP1-31140	Rabbit
*p*-Ret(Y905)_R_V	CST	3221	Rabbit
Slug(C19G7)_R_V	CST	9585	Rabbit
*p*-Smad2(S465/467)_R_V	CST	3101	Rabbit
SOCS1(A156)_R_V	CST	3950	Rabbit
SOCS3_R_V	CST	2923	Rabbit
Sox2(D6D9) XP_R_V	CST	3579	Rabbit
*p*-Src(Y527)_R_V	CST	2105	Rabbit
Stat1_R_V	CST	9172	Rabbit
*p*-Stat1(Y701)_R_V	CST	9171	Rabbit
*p*-Stat2(Y690)_R_V	CST	4441	Rabbit
*p*-Stat3(S727)_R_V	CST	9134	Rabbit
*p*-Stat4(Y693)_R_V	CST	5267	Rabbit
*p*-Stat5(Y694)_R_V	CST	9351	Rabbit
Stat6_R_V	CST	9362	Rabbit
*p*-Stat6(Y641)_R_V	CST	9361	Rabbit
*p*-Tuberin/TSC2(T1462)_R_V	CST	3611	Rabbit
Vimentin(D21H3)XP_R_V	CST	5741	Rabbit
Wnt5a/b(C27E8)_R_V	CST	2530	Rabbit
YAP(H125)_R_V	Santa Cruz Biotechnology	sc-15407	Rabbit
ZO-1_R_V	CST	5406	Rabbit
Caspase-7_R_V	CST	9491	Rabbit
VEGFR2(55B11)_R_V	CST	2479	Rabbit
Stat5a(L-20)_R_V	Santa Cruz Biotechnology	sc-1081	Rabbit
SOX9_R_V	EMD Millipore (Upstate)	AB5535	Rabbit
*p*-SAPK/JNK(T183/Y185)_R_V	CST	4668	Rabbit
Zeb1_R_V	Novus	NBP1-05987	Rabbit
PHF8(pAb)_R_V	Active Motif	39711	Rabbit
ASH2_R_V	Bethyl Laboratories	A300-489A	Rabbit
CBP(A-22)_R_V	Santa Cruz Biotechnology	sc-369	Rabbit
c-Fos(9F6) _R_V	CST	2250	Rabbit
CHAF1A(D77D5)XP_R_V	CST	5480	Rabbit
c-Jun(60A8)_R_V	CST	9165	Rabbit
CRSP1-TRAP220_R_V	Bethyl Laboratories	A300-793A	Rabbit
CtBP2_R_V	CST	13256	Rabbit
Cyclin C_R_V	Santa Cruz Biotechnology	sc-1061	Rabbit
DRIP130_R_V	Abcam	ab70450	Rabbit
FBX011_R_V	Abcam	ab72200	Rabbit
FoxK2_R_V	CST	12008	Rabbit
KLF4_R_V	CST	12173	Rabbit
MED12-Abcam_R_V	Abcam	ab70842	Rabbit
p300(C-20)_R_V	Santa Cruz Biotechnology	sc-585	Rabbit
*p*-c-Fos(S32)_R_V	CST	5348	Rabbit
PPP1R10(EPR11706)_R_V	Abcam	ab173285	Rabbit
Stat3(D3Z2G)_R_V	CST	12640	Rabbit
*p*-TRAP220-MED1(T1457)_R_V	Abcam	ab60950	Rabbit
HIF-2A(D9E3)_R_V	CST	7096	Rabbit
*p*-c-Jun(S63)_R_V	CST	9261	Rabbit
Sin3b_R_V	Abcam	ab101841	Rabbit
*p*-Rb(S807/811)_R_V	CST	9308	Rabbit
*p*-Rb(S780)(C84F6)_R_V	CST	3590	Rabbit
*p*-FAK(Y576/577)_R_V	CST	3281	Rabbit
Ki67_R_V	Vector Laboratories	VP-K451	Rabbit
Integrina4(D2E1)XP_R_V	CST	8440	Rabbit
Integrina5_R_V	CST	4705	Rabbit
IntegrinaV_R_V	CST	4711	Rabbit
Integrinb1(D2E5)_R_V	CST	9699	Rabbit
Integrinb3(D7X3P)XP_R_V	CST	13166	Rabbit
Integrinb4_R_V	CST	4707	Rabbit
PI3Kp85_R_V	CST	4292	Rabbit
AMPKa(23A3)_R_V	CST	2603	Rabbit
PI3Kp110a(C73F8)_R_V	CST	4249	Rabbit
ERa(SP1)_R_V	Thermo Fisher Scientific	RM-9101-S1	Rabbit
MEK6_R_V	Abcam	ab52937	Rabbit
p21_R_V	CST	2947	Rabbit
Atg12(D88H11)_R_V	CST	4180	Rabbit
Beclin-1(D40C5)_R_V	CST	3495	Rabbit
Caspase-3(Asp175)_R_V	CST	9661	Rabbit
Beta-Catenin(CT)_R_V	Invitrogen	71-2700	Rabbit
Atg3_R_V	CST	3415	Rabbit
Atg7_R_V	CST	8558	Rabbit
LC3A(D50G8)XP_R_V	CST	4599	Rabbit
LC3B(D11)XP_R_V	CST	3868	Rabbit
*p*-EGFR(Y845)_R_V	CST	2231	Rabbit
*p*-EGFR(Y992)_R_V	CST	2235	Rabbit
ErbB2/HER2(Y1248)_R_V	Imgenex	IMG-90189	Rabbit
*p*-SHC(2431)(Y317)_R_V	CST	2431	Rabbit
SRD5A1_R_V	Novusbio	H00006715-D01P	Rabbit
FAS_R_V	CST	3189	Rabbit
HexokinaseII(C64G5)_R_V	CST	2867	Rabbit
PFKFB3 (C-terminal)_R_V	Abcam	ab135820	Rabbit
RRM2 (EPR11820)_R_V	Abcam	ab172476	Rabbit
*p*-Stat1(Y701)(D4A7)_R_V	CST	7649	Rabbit
GLDC_R_V	CST	12794	Rabbit
GLS1_R_V	Proteintech	20170-1-AP	Rabbit
LDHA_R_V	CST	2012	Rabbit
PHGDH_R_V	CST	13428	Rabbit
PKM2_R_V	Bethyl Laboratories	A303-660A-M	Rabbit
PKM1/2_R_V	CST	3190	Rabbit
Bcl-xL_R_V	CST	2762	Rabbit
E-Cadherin(24E10)_R_V	CST	3195	Rabbit
ALK(D5F3)XP_R_V	CST	3633	Rabbit
HER2/c-ErbB2_R_V	Dako	A0485	Rabbit
ALDH_M_V	BD Biosciences	611194	Mouse
*p*-ATM(S1981)_M_V	CST	4526	Mouse
Axl_M_V	Abcam	ab54803	Mouse
CD24 Ab2(SN3b)_M_V	Thermo Fisher Scientific	MS-1279-P0	Mouse
Chk2(1C12)_M_V	CST	3440	Mouse
Cox-2_M_V	BD Biosciences	610203	Mouse
GATA3_M_V	BD Biosciences	558686	Mouse
GSK-3a/b_M_V	Santa Cruz Biotechnology	SC-7291	Mouse
HER2/c-ErbB2-P185_M_V	Invitrogen (BioSource)	AHO1011	Mouse
HIF-1a_M_V	BD Biosciences	610958	Mouse
*p*-IkappaB-a(S32/36)_M_V	BD Biosciences	551818	Mouse
S100A7/CBP_M_V	Abnova	H00006278-A01	Mouse
Twist(Twist2C1a)_M_V	Santa Cruz Biotechnology	sc-81417	Mouse
SRC-3(clone 1208/D1)_M_V	BCM-Mab	in house	Mouse
c-Myc(clone 1123)_M_V	BCM-Mab	in house	Mouse
SRC-2(TIF2)_M_V	BD	610985	Mouse
SRC-1(clone 1135/H4,1136/H4H6)_M_V	BCM-Mab	in house	Mouse
PR(1294)_M_V	Celetta	in house_Celetta	Mouse
Rb(4H1)_M_V	CST	9309	Mouse
*p*-FAK(Y397)_M_V	BD Biosciences	611806	Mouse
AR-441_M_V	BCM	NW-Mab70	Mouse
AMPKa(F6)_M_V	CST	2793	Mouse
Bcl2(Clone-124)_M_V	Dako	M088729-2	Mouse
c-Src(B-12)_M_V	Santa Cruz Biotechnology	sc-8056	Mouse
IGF-IR(3B7)_M_V	Santa Cruz Biotechnology	sc-462	Mouse
Laminin5_M_V	EMD Millipore	MAB19562	Mouse
Aromatase-A(Clone 677H7F10)_M_V	BCM-Mab	in house	Mouse
AOX1(cloneAO15)_M_V	Sigma-Aldrich	SAB4200562	Mouse
IDH2_M_V	Abcam	ab55271	Mouse
SCD1 (CD.E10)_M_V	Abcam	ab19862	Mouse

Host species for the antibodies and validation status are reported. R, rabbit; M, mouse and V, validated.

Fluorescence-labeled slides were scanned on a GenePix 4400 AL scanner. Each slide, along with its accompanying negative control slide, was scanned at an appropriate photomultiplier tube to obtain optimal signal for this specific set of samples. The images were analyzed with GenePix Pro 7.0 (Molecular Devices). Total fluorescence signal intensities for each spot were obtained after subtraction of the local background signal for each slide and were then normalized for variation in total protein, background, and nonspecific labeling using a group-based normalization method as described (Chang et al., 2015). Each image, along with its normalized data, was evaluated for quality through manual inspection and comparison with control samples. Specific fluorescence intensity signals of 50 units or less was taken as assay cutoff for a positive reaction, resulting in 195 remaining proteins, of the 212 (Table 1), to be analyzed.

### Analysis of data from RPPA assays

After data were normalized to PR signal in the assay or to target protein input, it was determined that normalizing to PR produced the most consistent results. The effects of receptor type and hormone treatment were analyzed via two-way ANOVA using R. We identified proteins with FDR-adjusted *p*-values <0.05. For this subset of identified proteins, relative fold changes between the liganded and unliganded receptor were calculated for each isoform. For proteins displaying a positive interaction with the liganded PR, a two-fold change was used as a cutoff. For proteins showing a positive association with the unliganded PR, further stringency was maintained by setting a higher threshold of three-fold change, as with the MS data.

### Western blot analysis

Western blot analyses were performed on representative proteins: synapsin-I and synapsin-II (MS-identified) and SRC-1, SRC-2, FoxO1, MED12, c-Fos, and c-Jun (RPPA-identified) to validate findings from the MS and RPPA analyses. Western blots were conducted as described previously (Bless et al., 2014) with the modifications indicated below. For independent validation of the ligand-dependent interaction of PR with hypothalamic SRC-1 and SRC-2 determined by RPPA, protein homogenates from female mouse hypothalamus (*n* = 6, 5 mice per group) were prepared as described above. Pull-down samples were probed for SRC-1 and SRC-2 using a rabbit polyclonal antibody to 350–690 amino acids (aa) of human SRC-1 (1:250, #sc-8995, Santa Cruz, RRID:AB_2235896) and a rabbit polyclonal antibody to human SRC-2 (1:750, #PA1-86392, Fisher Scientific, RRID:AB_2103955), respectively. MED12, c-Fos and c-Jun were probed with rabbit polyclonal antibodies MED12 (1:200, #ab70842, Abcam), c-Fos (1:200, #5348, Cell Signaling Technology), and c-Jun (1:200, #9261, Cell Signaling Technology), respectively. Fluorescently labeled donkey anti-goat AF488 and donkey anti-rabbit AF647 were used for detection of SRC-1 and SRC-2 (1:10,000, Invitrogen), respectively. Immunoreactive bands were detected and relative optical densities were quantified using a GelDoc Imager and Image Lab Software (Bio-Rad). Two-way ANOVA followed by Tukey’s honestly significant difference was used (SPSS v.21) to examine the effect of ligand and receptor type. We identified differences with Tukey-adjusted *p*-values <0.05. In addition, validation of MS findings of synapsin-I and synapsin-II by Western blot was conducted using a rabbit polyclonal antibody to synapsin-I (1:500, #ab1543, Millipore, RRID:AB_2200400) and a mouse monoclonal antibody to synapsin-II (0.3μg/mL, #mabn1573, Millipore). Secondary antibodies donkey anti-rabbit AF647 and donkey anti-mouse AF488 (1:10,000, Invitrogen) were used for synapsin-I and synapsin-II, respectively.

In confirmation of findings by RPPA, interaction of PR with FoxO1 was analyzed by Western blot using a rabbit polyclonal FoxO1 antibody (1:250, #2880, CST, RRID:AB_2106495), the same as used for RPPA, followed by a donkey anti-rabbit AF647 secondary antibody (1:10,000, Invitrogen). For the detection of mouse GST-PR, a mouse monoclonal antibody directed against the N-terminal 165–534 aa of human PR-A and PR-B (PR 1294, 0.5 µg/mL), followed by a donkey anti-mouse AF488 secondary antibody (1:10,000, Invitrogen), was used.

### Neuronal cultures

Primary cortical neuronal cultures (mixed sex) were prepared from Sprague-Dawley rat E18 embryos as described previously (Srivastava et al., 2011). These cortical cultures display mature neuronal morphology (extensive arborizations and dendritic spines) and electrophysiological and cellular responses to synaptic activity, consistent with those seen in *in vivo*/*ex vivo* studies of mature neurons (Xie et al., 2007; Srivastava et al., 2012). Because our MS and RPPA data were collected from mature brain, cortical neurons, which recapitulate mature neuronal characteristics (Xie et al., 2007; Srivastava et al., 2012), were used in these studies.

Animals were habituated for 3 d before experimental procedures, compliant with the Home Office Animals (Scientific Procedures) Act, UK, 1986. Dissociated cells were plated onto 18-mm glass coverslips (no. 1.5; 0117580, Marienfeld-Superior), coated with poly-d-lysine (0.2 mg/ml, Sigma-Aldrich), at a density of 3 × 10^5^/well equal to 857/mm^2^ and cultured in feeding medium: neurobasal medium (21103049) supplemented with 2% B27 (17504044), 0.5 mm glutamine (25030024), and 1% penicillin:streptomycin (15070063; all reagents from Invitrogen). Neuron cultures were maintained in the presence of 200 μm d,l-amino-phosphonovalerate (d,l-APV, ab120004, Abcam) beginning on 4 d *in vitro* (DIV 4) to maintain neuronal health for long-term culturing and reduce cell death due to excessive Ca^2+^ cytotoxicity via overactive NMDA receptors (Srivastava et al., 2011). Half-medium changes were performed twice weekly until the desired age (DIV 23–25).

### Pharmacological treatment of neuronal cultures

All pharmacological treatments were performed on DIV 25–30 primary cortical neuronal cultures. Treatments were conducted in feeding medium: neurobasal medium (21103049) supplemented with 2% B27, 0.5 mm glutamine, and 1% penicillin:streptomycin in the presence of 200 μm d,l-APV. Progesterone (P4; Sigma-Aldrich, P6149) was dissolved in ethanol to a concentration of 31.8 mm, and 17β-estradiol (E2) was dissolved in dimethylsulfoxide to a final concentration of 1 mm. Both compounds were serially diluted to a 10× working concentration in feeding medium and applied directly to neuronal cultures. Final concentrations of P4 and E2 were 1 nm. Final concentration of solvent was <0.01%: vehicle control was made up of solvent lacking compound, diluted as test compounds. Neuronal cultures were treated for 24 h, washed in PBS, and fixed in 4% formaldehyde/4% sucrose PBS for 10 min at room temperature followed by incubation in methanol prechilled to –20°C for 10 min at 4°C. Fixed neuronal cultures were then permeabilized and blocked simultaneously (2% normal goat serum, 5425S, Sigma-Aldrich, and 0.2% Triton X-100) before incubation with the following primary antibodies overnight: PSD95 mouse monoclonal antibody (1:1000; clone K28/43; #73-028, NeuroMab, RRID:AB_10698024), synapsin-I rabbit polyclonal antibody (1:200; #5297, Cell Signaling Technologies), and MAP2 chicken polyclonal antibody (1:2000; #AB104896, Abcam). Cultures were subsequently incubated with Alexa Fluor anti-rabbit 488, anti-mouse 568, and anti-chicken 633 secondary antibodies (1:750; Invitrogen) the following day for 1 h at room temperature (Srivastava et al., 2011).

### Quantitative and statistical analysis of immunofluorescence

Confocal images of neurons were acquired with a Nikon A1-R confocal microscope using a 60× oil-immersion objective (NA 1.4; Nikon) as a *z*-series, *z*-step set to 0.5 µm. Two-dimensional maximum-projection reconstructions of images were generated, and morphometric analysis (puncta number and intensity) was conducted using MetaMorph software (Universal Imaging Corporation; Srivastava et al., 2011). Analyses of puncta were performed on spines from at least two dendrites (secondary or tertiary branches), totaling 100 µm, from each neuron. The linear density of each synaptic protein cluster was measured automatically using MetaMorph (Srivastava et al., 2011). Synaptic puncta were defined as synapsin-I puncta that contained PSD95 immunofluorescence greater than background; background fluorescence was the average background intensity from five regions of interest plus two standard deviations (Glynn and McAllister, 2006). Cultures that were directly compared were stained simultaneously and imaged with the same acquisition parameters. For each condition, 10–16 neurons from at least 3 separate experiments were used. Experiments were conducted blind to condition and on sister cultures.

All statistical analyses were performed in GraphPad. Differences in quantitative immunofluorescence were identified by a one-way ANOVA with Tukey correction for multiple comparisons. Error bars represent the standard error of the mean.

## Results

### Mass spectrometry

To detect proteins from female mouse hypothalamus that associated with mouse PR in the presence or absence of hormone, MS analysis was performed on pull-down samples using GST-tagged PR. This unbiased approach identified many hypothalamic proteins that interact with one or both PR isoforms in a ligand-dependent manner (Tables 2 and 2-1). MS-identified proteins were categorized into the following groups based on their functions: (a) synaptic structure and function, (b) signal transduction, (c) transcription, (d) translation, and (e) metabolism. The proteins associated with synaptic structure and function was the largest group, containing 61% (20 of 35) of the MS-identified proteins that interacted with ligand-bound PR. Subsets of these synaptic proteins associated with PR-A only (9 proteins), PR-B only (7), or both isoforms (4) in a ligand-dependent manner, strongly indicating a function for both PR isoforms in synaptic physiology. More than 18% (6 of 35) of the identified PR-interacting proteins are transcriptional regulators, with two-thirds associating with PR-B only and the remaining one-third associating with PR-A only. Proteins involved in signal transduction, including kinases, comprised 9% (3 of 35) of the MS-identified proteins that associated with PR in the presence of ligand. We also identified the translational regulators 40S ribosomal protein S9 and 26S protease regulatory subunit 6A that associated only with PR-B in the presence of R5020. On the other hand, proteins with a role in cellular metabolism identified by MS interacted with liganded PR-A only (Table 2). Finally, consistent with previous studies (Bagchi et al., 1991; Johnson and Toft, 1994), a large number of additional proteins interacted with unliganded PR-A, PR-B, or both (Table 2-1).

**Table 2.** Proteins from mouse hypothalamus, identified by MS analysis, associate with mouse PR in a ligand-dependent manner

**Synaptic structure and function**	UniProt symbol	Uniprot ID	PR-A (R5020/no ligand)	PR-B (R5020/no ligand)
Synapsin-I	SYN1	O88935	+	+
Synapsin-II	SYN2	Q64332	+	+
Synapsin-III	SYN3	Q8JZP2		+
Synaptogyrin-III	SNG3	Q8R191	+	
α-Synuclein	SYUA	O55042		+
Septin-5	SEPTM5	Q9Z2Q6		+
Desmoglein-4	Desmoglein-4	Q7TMD7	+	
Desmoplakin	DESP	E9Q557	+	
Junction plakoglobin	PLAK	Q02257	+	
Leucine-rich repeat-containing protein 15	LGR4	A2ARI4	+	
Hemoglobin subunit β-1	HBB1	P02088	+	
β-Actin-like protein 2	ACTBL	Q8BFZ3		+
Tubulin α-1A chain	TBA1A	P68369		+
Tubulin α-1B chain	TBA1B	P05213		+
Tubulin β-2A chain	TBB2A	Q7TMM9	+	+
Tubulin β-3 chain	TBB3	Q9ERD7	+	
Tubulin β-4A chain	TBB4A	Q9D6F9	+	
Tubulin β-4B chain	TBB4B	P68372	+	
Tubulin β-5 chain	TBB5	P99024	+	+
Tubulin β-6 chain	TBB6	Q922F4		+
**Signal transduction**				
Mitogen-activated protein kinase 1	MK01	P63085		+
14-3-3 protein ζ/δ	1433Z	P63101		+
Rab GDP dissociation inhibitor β	GDIB	Q61598		+
**Transcription**				
Elongation factor 1β	TIF1B	Q62318	+	
Histone H4	H4	P62806	+	
Brain acid soluble protein 1	A4	P12023		+
Heterogeneous nuclear ribonucleoprotein D-like	HNRDL	Q9Z130		+
Heterogeneous nuclear ribonucleoprotein U-like protein 1	HNRL1	Q8VDM6		+
Probable ATP-dependent RNA helicase DDX5	DDX5	Q61656		+
**Translation**				
26S protease regulatory subunit 6A	PRS6A	O88685		+
40S ribosomal protein S9	RS9	Q6ZWN5		+
**Metabolism**				
Creatine kinase B-type	KCRB	Q04447	+	
Phytanoyl-CoA hydroxylase-interacting protein-like	PHIPL	Q8BGT8	+	

Proteins associate with PR with R5020/no ligand at a ratio ≥2 and *p* < 0.05. Table 2-1 shows proteins identified by MS that associate with PR in the absence of ligand.

10.1523/ENEURO.0272-17.2017.t2-1 Table 2-1 Proteins that interact with mouse PR-A, mouse PR-B, or both isoforms in the absence of ligand, as identified by MS, with a ratio of no ligand/R5020 ≥3 and *p* < 0.05. Download Table 2-1, DOC file.

**Figure 1.**
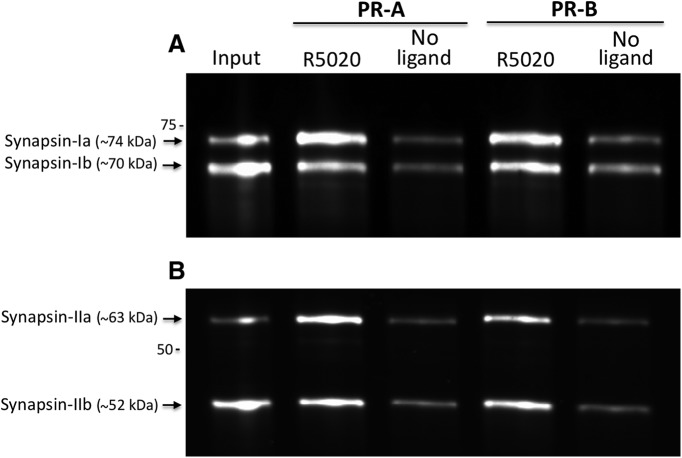
Hypothalamic synapsins interact with mouse PR-A and PR-B in a ligand-dependent manner. Confirmation by Western blot of MS findings that synapsin-Ia and synapsin-Ib (***A***) and synapsin-IIa and synapsin-IIb (***B***) associate with PR-A and PR-B in the presence, of the PR agonist R5020. Input (0.25% of total) from hypothalamic extracts (lane 1).

To validate findings by MS, pull-down samples were analyzed by Western blot for select synaptic proteins, the largest functional group identified in the MS analysis. Western blot results showed an increased interaction of synapsin-Ia and synapsin-Ib with both PR-A and PR-B in the presence of R5020 compared with no ligand (Fig. 1). Similarly, the synapsin-II isoforms, synapsin-IIa and synapsin-IIb, interacted with PR-A and PR-B in a ligand-dependent manner, further extending the MS findings.

### RPPA

To further explore the interactions of proteins from mouse hypothalamus with PR, we used RPPA, which provides a targeted approach and allows identification of phospho-proteins as markers of protein activity. RPPA analysis identified multiple proteins that interacted with mouse PR in a ligand-dependent and isoform-specific manner (Tables 3 and 3-1). In support of the present MS results, many of the identified proteins that associated with liganded PR were transcriptional regulators (Table 3). Additional proteins identified by RPPA that were not detected by MS have known roles in signal transduction or metabolism; a subset were phosphorylated forms of signaling proteins, including src kinase (Table 3).

**Table 3.** Mouse PR-A and PR-B interact with hypothalamic proteins, identified by RPPA, in a ligand-dependent manner, with a ratio of R5020/no ligand ≥2 and *p* < 0.05.

**Transcription**	UniProt symbol	UniProt ID	PR-A (R5020/no ligand)	PR-B (R5020/no ligand)
Steroid-receptor coactivator-1	NCOA1	P70365	+	+
Steroid receptor coactivator-2	NCOA2	Q61026	+	+
Thyroid hormone receptor-associated protein complex	MED12	A2AGH6	+	
c-Fos^p^	FOS	P01101	+	
c-Jun^p^	JUN	P05627	+	
Kruppel-like factor 4	KLF4	Q60793	+	
Autophagy-related protein 3	ATG3	Q9CPX6	+	
Autophagy related protein 12	ATG12	Q9CQY1	+	
BCL2 antagonist/killer 1	BAK	O08734	+	+
Forkhead box O1 (metabolism)	FOXO1	Q9R1E0	+	+
**Signal transduction**				
Src kinase^p^	SRC	P05480	+	
Signal transducer and activator of transcription 2^p^	STAT2	Q9WVL2	+	
Epidermal growth factor receptor	EGFR	Q01279	+	
p70S6 kinase^p^	KS6B	Q8BSK8	+	
Integrin-α5	ITA5	P11688	+	
Cyclin C	CCNC	Q62447	+	

^p^Proteins identified with phospho-specific antibodies. Table 3-1 shows proteins identified by RPPA that associate with PR in the absence of ligand.

**Figure 2.**
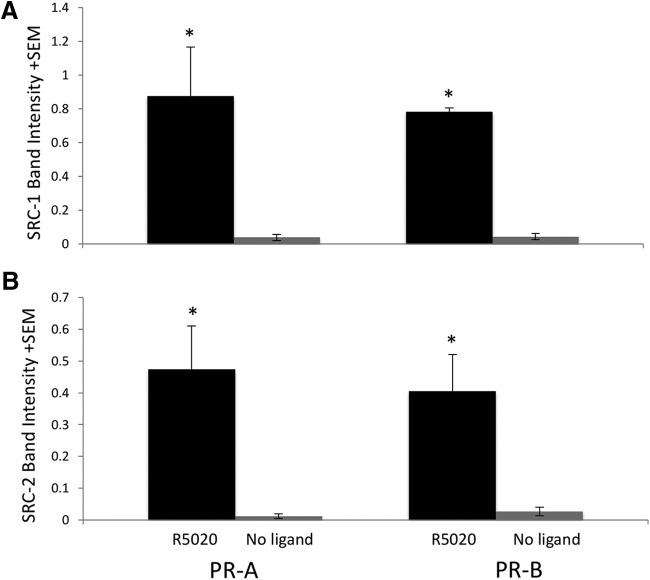
SRC-1 and SRC-2 from hypothalamus interact with PR in a ligand-dependent manner. Western blot results show that SRC-1 (***A***) and SRC-2 (***B***) from mouse hypothalamus associate with PR-A and PR-B in the presence of the agonist R5020, but not in the absence of ligand. *p < 0.05 indicates difference between the presence and absence of the ligand.

**Figure 3.**
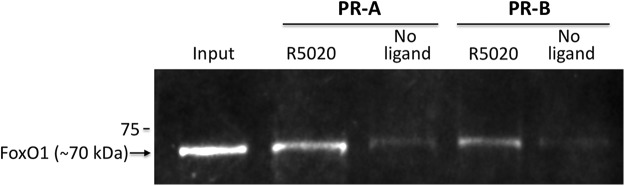
Hypothalamic FoxO1 interacts with mouse PR-A and PR-B when bound to the agonist, R5020. Confirmation of RPPA findings by Western blot that FoxO1 from mouse hypothalamus associates with PR-A and PR-B in the presence, but not the absence, of the PR agonist R5020. Inputs (1% of total) of FoxO1 from hypothalamic extracts (lane 1).

Two nuclear receptor coactivators, SRC-1 and SRC-2, known to be involved in PR expression and function in brain (Molenda et al., 2002; Molenda-Figueira et al., 2006, 2008; Yore et al., 2010; Acharya et al., 2015), were also detected by RPPA to interact with PR-A and PR-B in the presence of R5020 compared with no ligand. Ligand-dependent interactions with SRC-1 and SRC-2 were further confirmed by Western blot analysis (Fig. 2). Two-way ANOVA on SRC-1 relative intensity showed an effect of ligand (*F* = 14.56, *p* = 10.0 × 10^–4^) such that SRC-1 interacted with PR-A and PR-B in the presence of R5020 compared with no ligand. Similarly, SRC-2 associated with both PR isoforms when bound to R5020 (*F* = 21.65, *p* = 3.0 × 10^–5^) compared with no ligand. There were differences between receptor subtypes on interaction with either SRC-1 or SRC-2 (Fig. 2). Interestingly, the majority of proteins that function as transcriptional regulators, as identified by RPPA, interacted with PR-A only (Table 3). Furthermore, proteins involved in signal transduction identified by RPPA interacted with PR-A, but not PR-B, in the presence of agonist (Table 3). A transcription factor with a role in energy metabolism, FoxO1, was also identified to interact with both PR-A and PR-B. This interaction of FoxO1 with PR was further validated by Western blot (Fig. 3). Confirmation of c-Fos, c-Jun, and MED12, identified by RPPA, by Western blot was not possible because the signal intensities of these proteins by Western were not strong enough to be analyzed, most likely because of the decreased sensitivity of Western blot compared with RPPA. Two other proteins identified by MS to interact with liganded PR, MAP kinase 1 and 14-3-3 protein ζ/δ, were not found to associate with PR in a ligand-dependent manner by RPPA, likely because of differences in sensitivities for specific proteins between the two techniques.

Similar to the MS results above, additional proteins that selectively associated with unliganded PR compared with their liganded counterparts were identified by RPPA (Table 3-1).

10.1523/ENEURO.0272-17.2017.t3-1 Table 3-1 Proteins that interact with mouse PR-A, mouse PR-B, or both isoforms in the absence of a ligand, as identified by RPPA, with a ratio of no ligand/R5020 ≥3 and *p*<0.05. ^p^Proteins identified with phospho-specific antibodies. Download Table 3-1, DOC file.

### Ingenuity pathway analysis of PR interactome

**Figure 4.**
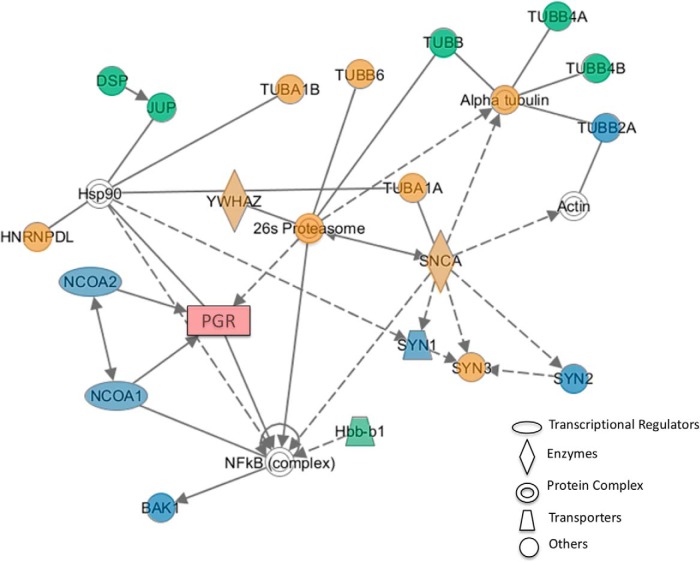
Network generated by Ingenuity Pathway Analysis (IPA) showing proteins from hypothalamus that associate with liganded PR-A and PR-B. Shaded proteins were identified to associate with both PR-A and PR-B (blue), PR-A only (green), or PR-B only (orange) in the present study. Unshaded proteins (white) were added to the network based on curated relationships in the IPA database. BAK1, BCL2 antagonist/killer 1; DSP, desmoplakin; JUP, junction plakoglobin; Hbbb1, hemoglobin subunit β-1; HNRNPDL, heterogeneous nuclear ribonucleoprotein D-like; NCOA1/SRC-1, steroid receptor coactivator-1; NCOA2/SRC-2, steroid receptor coactivator-2; PGR, progesterone receptor; SNCA, α-synuclein; SYN, synapsin; TUB, tubulin; YWHAZ, 14-3-3 protein ζ/δ.

Network pathways of proteins that associate with PR, as identified by MS and RPPA, were created using Ingenuity Pathway Analysis, a program that generates protein networks using known protein interactions (Fig. 4). The highest scoring network pathway depicted shows proteins known to associate with PR (e.g., NCOA1 and NCOA2) and novel interactions of proteins from brain (e.g., synapsins and tubulin complexes) with PR identified in the present study (Fig. 4).

The use of the two proteomics approaches, MS and RPPA, enabled a more comprehensive identification of novel and known proteins that interacted with PR-A and PR-B in a ligand-dependent manner. Although MS analysis of PR-protein pull-down assays is an unbiased approach, it is less sensitive than RPPA for detection of lower-abundance proteins such as SRC-1 and SRC-2. RPPA, on the other hand, is an antibody-based targeted proteomic approach and is limited to the target antigens for which antibodies are available. For example, our RPPA platform does not have validated antibodies to synaptic proteins, the largest functional groups identified by MS. Therefore, MS and RPPA served as complementary approaches for identification of PR-interacting proteins.

### Effects of progesterone and estradiol on synapsin-I– and PSD95-containing synapses

**Figure 5.**
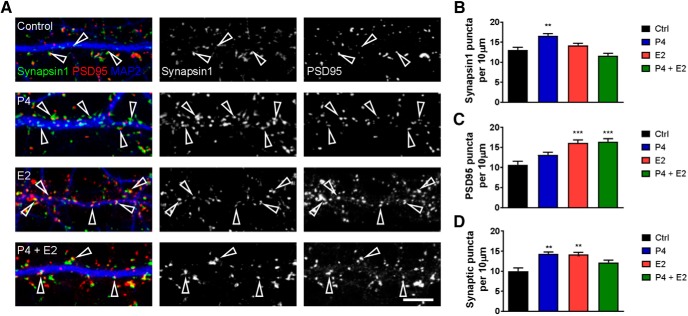
Progesterone (P4) and estradiol (E2) alter synapse number and synaptic protein expression. ***A***, Representative confocal images of primary rat cortical neurons treated with 1 nm P4, 1 nm E2, or both for 24 h. Images are for synapsin-I (green) and PSD95 (red) along MAP2-positive dendrites (blue). Open arrowheads indicate colocalized synapsin-I and PSD95 puncta and thus, synapses. ***B***, Quantitative analysis of synapsin-I linear density; treatment with P4 increased synapsin-I density compared with control (vehicle) condition. ***C***, PSD95 linear density was increased over control levels after treatment with E2 alone or combined P4 and E2 treatment. ***D***, Measurement of synaptic puncta, defined as synapsin-I puncta positive for PSD95, was increased after treatment with either P4 or E2. **p < 0.01; ***p < 0.001; scale bar = 5 µm.

To understand whether P4 could alter synapsin-I levels, primary rat cortical neurons with a mature morphology (DIV 23–25) were treated with 1 nm P4, 1 nm E2, or 1 nm P4 and E2 for 24 h. Subsequently, cells were fixed and immunostained for synapsin-I, postsynaptic density protein 95 (PSD95), and MAP2 (marker of neuronal morphology). Quantitative analysis of immunofluorescence revealed that after exposure to P4, there was an increase in the linear density of synapsin-I puncta (Fig. 5*A*, *B*). E2, on the other hand, did not alter synapsin-I linear density from control (vehicle) levels; the combined treatment of P4 and E2 also had no effect on synapsin-I levels (synapsin-I puncta linear density per 10 µm: control, 13.0 ± 0.71; P4, 16.6 ± 0.53; E2, 14.2 ± 0.49; P4 + E2, 11.6 ± 0.58; Fig. 5*A*, *B*). When we examined levels of the postsynaptic protein PSD95, no change in linear density was observed in cells treated with P4. However, an increase in the PSD95 linear density was observed in cells treated with E2 or P4 plus E2 (PSD95 puncta linear density per 10 µm: control, 10.7 ± 0.85; P4, 13.1 ± 0.69; E2, 16.6 ± 0.68; P4 + E2, 16.4 ± 0.74; Fig. 5*A*, *C*). We next sought to determine whether the changes in synaptic protein linear density induced by P4, E2, or P4 plus E2 treatments reflected an overall change in synapse number; synapses were defined as synapsin-I puncta overlapping with PSD95. In line with the observed increases in synapsin-I and PSD95 linear density in P4- and E2-treated cells, respectively, an increase in synapse number was also observed, whereas the combined treatment of P4 and E2 did not alter synapse number from control levels (synaptic puncta linear density per 10 µm: control, 9.9 ± 0.80; P4, 14.3 ± 0.49; E2, 14.2 ± 0.51; P4 + E2, 12.15 ± 0.59; Fig. 5*A*, *D*).

## Discussion

Using the proteomics-based approaches of MS and RPPA, we have identified proteins from female mouse hypothalamus that form complexes with PR-A, PR-B, or both in a ligand-dependent manner. Of the proteins that associated with agonist-bound PR, synaptic modulators made up the largest group, providing evidence for novel roles of PR in synaptic plasticity. Furthermore, in primary cortical neuronal cultures, we provide evidence that progesterone increases synapses containing synapsin-I protein, which selectively associated with ligand-bound PR. The other major protein groups from hypothalamus that interacted with the PR isoforms are kinases and signaling proteins, transcriptional and translational regulators, and proteins linked to energy metabolism.

### PR and synaptic modulation

Progestins modulate dendritic spine density and synapse numbers in the female rat hippocampus, ventromedial hypothalamus (VMH), and prefrontal cortex, and in cultured rat cortical neurons (Woolley and McEwen, 1993; McEwen and Woolley, 1994; Chen et al., 2009; Griffin et al., 2010; Chisholm and Juraska, 2012; Sanchez et al., 2013). Most prior studies have examined the combined effects of E2 and P4 on dendritic spines, revealing that E2 increases, whereas E2 plus P4 decreases, dendritic spines in the hippocampus (McEwen and Woolley, 1994; Murphy and Segal, 2000), prefrontal cortex (Chisholm and Juraska, 2012), and hypothalamus (Griffin et al., 2010). Previously, it has been shown that P4 treatment for 2 wks increases dendritic spine density in cortical pyramidal neurons of ovariectomized rats (Chen et al., 2009). The present findings extended those of Chen et al. (2009) to reveal that treatment with P4 for 24 h increases synapses in cortical neurons, as indicated by immunohistochemical localization of synapsin-I and PSD95 in the newly formed synapses. In further support of progestin-dependent synapse modulation, estradiol-induced PR have been detected in neuronal processes in the hypothalamus (Blaustein et al., 1988) and dendritic spines and axon terminals in the hippocampus (Waters et al., 2008; Mitterling et al., 2010) of female rodents.

The present findings, that synaptic proteins from mouse brain interact with PR, provide insight into novel mechanisms for progestin effects on synaptic functions. As identified by MS and Western blot, synapsin-I and synapsin-II interacted with both PR isoforms in a ligand-dependent manner. In addition, P4 increased synaptic density in rat primary cortical neurons, indicated by an increase in synapsin-I–positive puncta, suggesting that synapsin-I function in P4-mediated synapse addition is conserved among species. The present findings confirm the synaptogenic role of progestins and suggest a function for synapsin-I in progestin-mediated synapse formation. Synapsins may influence cortical synapses through direct association with PR or, alternatively, may function with PR indirectly or through PR-independent pathways.

Synapsins, including synapsin-I and synapsin-II, are presynaptic phosphoproteins that are integral in axon formation, synaptic maturation, transmission, and remodeling in excitatory synapses (Chin et al., 1995; Ferreira et al., 1998; Bogen et al., 2009; Cesca et al., 2010). Synapsins contain binding sites for MAPK/Erk and src kinase (Jovanovic et al., 1996; Foster-Barber and Bishop, 1998), kinases identified in the present study to interact with ligand-bound PR. Taken together, these findings suggest that PR increase synapses through regulating expression, transport, or phosphorylation of synapsins. Given the rapid effects of estrogens on synapses via kinases and synaptic proteins (Srivastava et al., 2008; Sellers et al., 2015), it will be important to explore the function of kinases identified here on progestin-mediated synapse formation.

Multiple synaptic proteins interacted with PR in an isoform-specific manner. For example, synaptogyrin-III, which is upregulated in the hypothalamus by estradiol (Malyala et al., 2005), interacted only with PR-A, suggesting a role for this protein in hormone-dependent synaptic changes. Synapsin-III, which is expressed in cell bodies of neuronal progenitors and has been implicated in neurogenesis (Kao et al., 2008), interacted only with PR-B, indicating a role for PR-B in neurogenesis (Barha et al., 2011; Bali et al., 2012). Interestingly, tubulins, which are components of microtubules that provide structural framework for synaptic junctions, postsynaptic densities, and synaptic vesicles (Kelly and Cotman, 1978; Zisapel et al., 1980), formed complexes with PR in an isoform-specific and ligand-dependent manner, another novel finding of the current study. Tubulinβ-2A and tubulinβ-4B, which are upregulated by estradiol in female hippocampus, were associated with liganded PR in the present study (Pechenino and Frick, 2009; Sárvári et al., 2015). Taken together, these findings suggest an important role for tubulins in progestin-mediated synaptic plasticity in the brain.

### PR and signal transduction

PR, acting through kinase activation, exert rapid effects in the brain (Mani et al., 2006; Tetel, 2009; Sanchez et al., 2013). In further support of rapid effects of PR in the brain, we found that MAPK1 associated with liganded PR-B, but not PR-A. Consistent with these findings, MAPK1 associates with and phosphorylates PR-B, but not PR-A, in P4-treated breast cancer cells (Clemm et al., 2000; Boonyaratanakornkit et al., 2007). In addition, we detected hormone-dependent interactions between src kinase and EGFR with PR-A, but not PR-B. These differential interactions of signaling molecules with PR may be important for isoform-specific rapid effects of PR in brain.

### PR and energy metabolism

Although the profound effects of estradiol on decreasing feeding and weight gain and increasing activity in women and female rodents is well known (Blaustein and Wade, 1976; Clegg, 2012; Bless et al., 2014, 2016), the effects of progesterone on metabolism are less studied. However, progesterone attenuates the effects of estrogens on food intake and carbohydrate metabolism in female primates and women, respectively (Kemnitz et al., 1989; D’Eon et al., 2002; Michopoulos and Wilson, 2011). The present findings reveal that creatine kinase B and phytanoyl COA-hydroxylase interacting protein-like, both involved in energy metabolism (James et al., 2012; Schlattner et al., 2016), associated with PR-A, but not PR-B, implicating a function for hypothalamic PR-A in energy homeostasis.

In further support of PR function in energy homeostasis, the transcriptional regulator FoxO1 interacted with both PR isoforms. FoxO1 is expressed in energy regulation centers, including the VMH and arcuate nucleus (Kim et al., 2006; Fukuda et al., 2008), and enhances food intake by increasing neuropeptide Y and agouti-related protein mRNA expression (Kitamura et al., 2006; Huang et al., 2012). In addition, FoxO1 attenuates leptin signaling and is regulated by leptin (Yang et al., 2009; Ren et al., 2013). Taken together with the studies above, the present findings suggest that hypothalamic PR function in energy homeostasis and indicate the need for further study of progestins in food intake and energy metabolism.

### PR and transcriptional regulation

PR-A and PR-B act as transcription factors in concert with other transcriptional regulators, including nuclear receptor coactivators and RNA polymerases (O’Malley et al., 2012; Tetel and Acharya, 2013; Dasgupta et al., 2014). The present findings reveal that SRC-1 and SRC-2 from hypothalamus interact with agonist-bound PR-A and PR-B, consistent with our earlier work that these coactivators are coexpressed with the PR isoforms in the female mouse hypothalamus (Acharya et al., 2015) and function in the brain to modulate PR-dependent behaviors (Molenda-Figueira et al., 2006).

Although the role of the transcription factor FoxO1 in energy metabolism is discussed above, it has also been linked to stress and depression. FoxO1 expression is upregulated by glucocorticoids (Qin et al., 2014) and is downregulated by antidepressants (Polter et al., 2009). Thus, exploring a potential role of FoxO1 and PR interaction may provide more insight into the role of progesterone in stress disorders.

PR-A, but not PR-B, interacted with MED12, which bridges transcription factors with RNA polymerases and is integral for vertebrate neuronal development (Wang et al., 2006; Rocha et al., 2010; Kim et al., 2016). In humans, MED12 mutations lead to X-linked mental retardation (Philibert et al., 2001; Risheg et al., 2007; Schwartz et al., 2007). Taken together, the present findings provide a mechanism by which progestins mediate brain development (Quadros et al., 2008; López and Wagner, 2009) through PRA-MED12 interactions. In future studies, it will be important to explore whether mutations in MED12 disrupt interactions with PR and alter brain development.

Based on previous studies and the current findings, a majority of tubulins and synaptic proteins, including synapsin isoforms and α-synuclein, likely associate with PR through a 26S proteasome complex as shown by Ingenuity Pathway Analysis (Fig. 4). Interestingly, 26S protease subunit associated with PR-B, but not PR-A, in the current study, suggesting differential downregulation of the PR isoforms after P4 treatment (Lange et al., 2000). Ingenuity Pathway Analysis incorporates previously identified proteins that are known to associate with PR, in either the liganded or unliganded state, with the present findings. For example, proteins known from previous studies to interact with PR, including a molecular chaperone hsp90 (DeMarzo et al., 1991; Johnson et al., 1994; Tetel et al., 1997), actin (known for its role in PR- mediated growth cone formation and modulation of dendritic spines; Sanchez et al., 2013; Wessel et al., 2014), and nuclear factor-κB (a stress-response regulator; Cutolo et al., 2004), were added to the network (Fig. 5) by Ingenuity Pathway Analysis. However, given that the present study focused on hypothalamic proteins that interact with PR in a ligand-dependent manner, only these proteins from the current findings were added to the network (shaded proteins in Fig. 5). In summary, this network confirms previously known interactions of PR with nuclear receptor coactivators (e.g., SRC-1/NCOA1 and SRC-2/NCOA2) and provides evidence for novel mechanisms by which progestins modulate synapses, revealing multiple regulatory levels of PR action in the brain.

Although genome-wide analysis of PR binding sites in mouse uterus (Rubel et al., 2012) and computational models have been used to predict the human PR interactome (Liu et al., 2015), to the best of our knowledge, the PR interactome has not been previously analyzed. Despite different approaches used in the previous studies (Rubel et al., 2012; Liu et al., 2015), the present findings of association of PR with transcriptional regulators, multiple tubulin isoforms, Src kinase, and EGFR were consistent with these published reports. Other studies have focused on receptor-dependent transcriptome or proteome of steroid receptors, including PR and estrogen receptors (Tan et al., 2012; Yang et al., 2017). Finally, proximity mapping has been used in cultured cells to identify proteins showing ligand-dependent interactions with receptors for glucocorticoids and androgens (Lempiäinen et al., 2017). Although these studies have found some interacting partners similar to those of the present study, including SRC-1, SRC-2, and MED12, none of these experiments investigated interacting proteins from the brain, and more specifically the hypothalamus. Therefore, the current study has identified a number of novel proteins interacting with PR, including synaptic proteins and regulators of metabolism.

A comprehensive and novel approach of combining MS and RPPA enabled us to identify a diverse and new group of protein complexes in the brain that interact with PR in a ligand- and isoform-specific manner. Although MS is an unbiased approach for identification of novel proteins, it requires large amounts of protein input, which could result in difficulty identifying low-abundance proteins. RPPA complemented the MS and expanded the coverage for low-abundance proteins. Both approaches identified many proteins in three major groups, including those with roles in transcription, metabolism, and signal transduction. In future studies, it will be important to investigate interactions between endogenous hypothalamic PR with proteins using different approaches (e.g., coimmunoprecipitation assays) to confirm and extend the current findings.

Through the identification of multiple components of PR complexes in the brain, the current study provides insights into various mechanisms of PR in physiology and disease. In addition, identification of protein complexes from female mouse hypothalamus that interact differentially with PR-A and PR-B sheds light on mechanisms that may contribute to PR isoform–specific functions. This study provides a novel role for synapsin-I in progestin-induced increase in synapses. These findings offer further evidence for overlapping pathways in genomic and nongenomic regulation in synaptic physiology and energy metabolism. In future studies, it will be important to apply the present strategies to identify factors involved in ligand-independent activation of PR (Denner et al., 1990; Mani et al., 1994; Tetel and Lange, 2009) that will provide important insights into PR action in physiology, behavior, and disease.

## Synthesis

Reviewing Editor: Jeffrey Blaustein, University of Massachusetts Decisions are customarily a result of the Reviewing Editor and the peer reviewers coming together and discussing their recommendations until a consensus is reached. When revisions are invited, a fact-based synthesis statement explaining their decision and outlining what is needed to prepare a revision will be listed below. The following reviewer(s) agreed to reveal their identity: Robert Handa

There is great interest in your manuscript, in particular identifying the protein partners of PR in the hypothalamus, and everybody is in agreement that the work should be published. However, there are some issues that will need to be dealt with:

1. Please remove reference to your study being “the first study to provide a progestin-dependent proteomic ...” Besides the fact that one can never know that they are first to do something, one reviewer points out that, while no one else may have approached this question in precisely the same way previously, the claim is perhaps stronger than is warranted, based on the literature and the data presented.

2. Questions were raised in the first review of this MS regarding the novelty of the findings, given that previous studies in both neural and non-neural tissues have demonstrated some of the protein interactions shown here (Reviewer 1 “the fact that in mouse there are now similar findings is not all that groundbreaking”). While not as complete as the present study, previous work (by Edwards for example) in non-neural tissues demonstrated some of the kinase interactions reported here, while papers cited in the reference list demonstrated receptor subtype specific interactions with steroid receptor coactivator-1 and -2 (Molenda-Figueira et al Endocrinology 2008; Yore et al Neuroscience 2010) consistent with the observations reported here. That progesterone may promote synapse formation in the cortex was suggested eight years ago by the work of Chen at al (Cereb Cortex. 2009; a paper that is not in the reference list) who reported that progesterone alone was as effective as estradiol in restoring the dendritic spine densities of primary sensorimotor cortical pyramidal neurons, in ovariectomized rats.

3. It was suggested that the cortical culture addition actually detracts from the paper, and that, the paper of Chen et al., could simply be referred to. That is your decision, but it is most important to focus on the protein interaction data in the hypothalamus, which are definitely worthy of publication. Either way, it is essential to tone down the discussion of cortical spines, and address directly in the discussion that this was done in cortex, not hypothalamus.
