# Supplementary material for: The Progestin Receptor Interactome in the Female Mouse Hypothalamus: Interactions with Synaptic Proteins Are Isoform Specific and Ligand Dependent
Source: eNeuro. 2017 Sep 20;4(5):ENEURO.0272-17.2017. doi: 10.1523/ENEURO.0272-17.2017 (PMC5605756; doi:10.1523/ENEURO.0272-17.2017)
Supplement: Table 2-1 [file enu005172413so3.doc]

|  | **Table 2-1** |  |  |  |  |  |  |  |
| --- | --- | --- | --- | --- | --- | --- | --- | --- |
|  | **Protein Name** | **UniProt Symbol** | **UniProt ID** | **PR-A (no ligand/ R5020)** | **PR-B (no ligand/ R5020)** |  |  |  |
|  | 1-phosphatidylinositol 4,5-bisphosphate phosphodiesterase β1 | PLCB1 | Q9Z1B3 | + | + |  |  |  |
|  | 60S ribosomal protein L4 | RL4 | Q9D8E6 | + | + |  |  |  |
|  | 60S ribosomal protein L8 | RL8 | P62918 | + | + |  |  |  |
|  | 60S ribosomal protein L18 | RL18 | P35980 | + | + |  |  |  |
|  | 60S ribosomal protein L19 | RL19 | P84099 | + | + |  |  |  |
|  | Heat shock 70 kDa protein 4 | HSP74 | Q61316 | + | + |  |  |  |
|  | Nucleolar transcription factor 1 | UBF1 | P25976 | + | + |  |  |  |
|  | Nucleolin | NUCL | P09405 | + | + |  |  |  |
|  | Peroxiredoxin-4 | PRDX4 | O08807 | + | + |  |  |  |
|  | Serine/threonine-protein phosphatase 2B catalytic subunit α | PP2BA | P63328 | + | + |  |  |  |
|  | Stress-70 protein, mitochondrial | GRP75 | P38647 | + | + |  |  |  |
|  | Syntaxin-binding protein 1 | STXB1 | O08599 | + | + |  |  |  |
|  | Ubiquitin-conjugating enzyme E2 O | UBE2O | Q6ZPJ3 | + | + |  |  |  |
|  | 40S ribosomal protein S23 | RS23 | P62267 | + |  |  |  |  |
|  | 40S ribosomal protein S8 | RS8 | P62242 | + |  |  |  |  |
|  | 60S ribosomal protein L3 | RL3 | P27659 | + |  |  |  |  |
|  | 60S ribosomal protein L7 | RL7 | P14148 | + |  |  |  |  |
|  | Brain acid soluble protein 1 | BASP1 | Q91XV3 | + |  |  |  |  |
|  | Breast carcinoma-amplified sequence 1 homolog | BCAS1 | Q80YN3 | + |  |  |  |  |
|  | Complement C1q subcomponent subunit B | C1QB | P14106 | + |  |  |  |  |
|  | DNA topoisomerase 2β | TOP2B | Q64511 | + |  |  |  |  |
|  | Histone H1.4 | H14 | P43274 | + |  |  |  |  |
|  | Ras-related protein Rab-12 | RAB12 | P35283 | + |  |  |  |  |
|  | RNA-binding protein EWS | EWS | Q61545 | + |  |  |  |  |
|  | RNA-binding protein FUS | FUS | P56959 | + |  |  |  |  |
|  | Splicing factor, proline- and glutamine-rich | SFPQ | Q8VIJ6 | + |  |  |  |  |
|  | Splicing factor, proline- and glutamine-rich | SFPQ | Q8VIJ6 | + |  |  |  |  |
|  | Treacle protein | TCOF | O08784 | + |  |  |  |  |
|  | 6-phosphofructokinase type C | PFKAP | Q9WUA3 |  | + |  |  |  |
|  | 6-phosphofructokinase, liver type | PFKAL | P12382 |  | + |  |  |  |
|  | 60S ribosomal protein L26 | RL26 | P61255 |  | + |  |  |  |
|  | 78 kDa glucose-regulated protein | GRP78 | P20029 |  | + |  |  |  |
|  | Aconitate hydratase, mitochondrial | ACON | Q99KI0 |  | + |  |  |  |
|  | Actin-related protein 2/3 complex subunit 1A | ARC1A | Q9R0Q6 |  | + |  |  |  |
|  | AP-2 complex subunit α-2 | AP2A2 | P17427 |  | + |  |  |  |
|  | Aspartate aminotransferase, cytoplasmic | AATC | P05201 |  | + |  |  |  |
|  | ATP synthase subunit β, mitochondrial | ATPB | P56480 |  | + |  |  |  |
|  | ATP-dependent RNA helicase DDX3X | DDX3X | [Q62167](http://www.uniprot.org/uniprot/Q62167) |  | + |  |  |  |
|  | Brevican core protein | PGCB | Q61361 |  | + |  |  |  |
|  | Calcium/calmodulin-dependent protein kinase type II-α | KCC2A | [P11798](http://www.uniprot.org/uniprot/P11798) |  | + |  |  |  |
|  | Calcium/calmodulin-dependent protein kinase type II-β | KCC2B | P28652 |  | + |  |  |  |
|  | CaM kinase-like vesicle-associated protein | CAMKV | Q3UHL1 |  | + |  |  |  |
|  | Cyclin-dependent kinase 12 | CDK12 | Q14AX6 |  | + |  |  |  |
|  | Cytoplasmic FMR1-interacting protein 1 | CYFP1 | Q7TMB8 |  | + |  |  |  |
|  | Cytosolic acyl coenzyme A thioester hydrolase | BACH | Q91V12 |  | + |  |  |  |
|  | D-3-phosphoglycerate dehydrogenase | SERA | Q61753 |  | + |  |  |  |
|  | Dihydropyrimidinase-related protein 4 | DPYL4 | O35098 |  | + |  |  |  |
|  | Dihydropyrimidinase-related protein 5 | DPYL5 | Q9EQF6 |  | + |  |  |  |
|  | Elongation factor 2 | EF2 | P58252 |  | + |  |  |  |
|  | Fructose-bisphosphate aldolase C | ALDOC | P05063 |  | + |  |  |  |
|  | Glutamate dehydrogenase 1, mitochondrial | DHE3 | P26443 |  | + |  |  |  |
|  | Glycogen synthase kinase-3 β | GSK3B | Q9WV60 |  | + |  |  |  |
|  | Guanine deaminase | GUAD | Q9R111 |  | + |  |  |  |
|  | Heat shock 70 kDa protein 1-like | HS71L | P16627 |  | + |  |  |  |
|  | Heat shock 70 kDa protein 12A | HS12A | Q8K0U4 |  | + |  |  |  |
|  | Heat shock 70 kDa protein 1A | HS71A | Q61696 |  | + |  |  |  |
|  | Heat shock 70 kDa protein 4L | HS74L | P48722 |  | + |  |  |  |
|  | Heat shock cognate 71 kDa protein | HSP7C | P63017 |  | + |  |  |  |
|  | Heterogeneous nuclear ribonucleoprotein A3 | ROA3 | Q8BG05 |  | + |  |  |  |
|  | Heterogeneous nuclear ribonucleoprotein H | HNRH1 | O35737 |  | + |  |  |  |
|  | Heterogeneous nuclear ribonucleoprotein K | HNRPK | P61979 |  | + |  |  |  |
|  | Heterogeneous nuclear ribonucleoproteins A2/B1 | ROA2 | O88569 |  | + |  |  |  |
|  | Histone-binding protein RBBP4 | RBBP4 | Q60972 |  | + |  |  |  |
|  | L-lactate dehydrogenase A chain | LDHA | P06151 |  | + |  |  |  |
|  | LanC-like protein 1 | LANC1 | O89112 |  | + |  |  |  |
|  | Myelin proteolipid protein | MYPR | P60202 |  | + |  |  |  |
|  | N-acylneuraminate cytidylyltransferase | NEUA | Q99KK2 |  | + |  |  |  |
|  | Non-POU domain-containing octamer-binding protein | NONO | [Q99K48](http://www.uniprot.org/uniprot/Q99K48) |  | + |  |  |  |
|  | Peroxiredoxin-1 | PRDX1 | P35700 |  | + |  |  |  |
|  | Peroxiredoxin-2 | PRDX2 | Q61171 |  | + |  |  |  |
|  | Phosphoglycerate kinase 1 | PGK1 | [P09411](http://www.uniprot.org/uniprot/P09411) |  | + |  |  |  |
|  | Phytanoyl-CoA hydroxylase-interacting protein | PHYIP | Q8K0S0 |  | + |  |  |  |
|  | Phytanoyl-CoA hydroxylase-interacting protein-like | PHIPL | Q8BGT8 |  | + |  |  |  |
|  | Poly(rC)-binding protein 1 | PCBP1 | P60335 |  | + |  |  |  |
|  | Potassium-transporting ATPase α chain 2 | AT12A | Q9Z1W8 |  | + |  |  |  |
|  | Profilin-2 | PROF2 | Q9JJV2 |  | + |  |  |  |
|  | Rab GDP dissociation inhibitor α | GDIA | P50396 |  | + |  |  |  |
|  | Ras-related protein Rab-10 | RAB10 | P61027 |  | + |  |  |  |
|  | Rho GDP-dissociation inhibitor 1 | GDIR1 | Q99PT1 |  | + |  |  |  |
|  | Serine/threonine-protein phosphatase 2B catalytic subunit β | PP2BB | P48453 |  | + |  |  |  |
|  | Sodium/potassium-transporting ATPase subunit α-1 | AT1A1 | Q8VDN2 |  | + |  |  |  |
|  | Sodium/potassium-transporting ATPase subunit α-3 | AT1A3 | [Q6PIC6](http://www.uniprot.org/uniprot/Q6PIC6) |  | + |  |  |  |
|  | Synaptogyrin-3 | SNG3 | Q8R191 |  | + |  |  |  |
|  | Synaptojanin-1 | SYNJ1 | Q8CHC4 |  | + |  |  |  |
|  | Synaptotagmin-1 | SYT1 | P46096 |  | + |  |  |  |
|  | Transcriptional activator protein Pur-α | PURA | P42669 |  | + |  |  |  |
|  | Ubiquitin-like modifier-activating enzyme 1 | UBA1 | [Q02053](http://www.uniprot.org/uniprot/Q02053) |  | + |  |  |  |
|  | V-type proton ATPase 116 kDa subunit a isoform 1 | VPP1 | Q9Z1G4 |  | + |  |  |  |
|  | V-type proton ATPase catalytic subunit A | VATA | [P50516](http://www.uniprot.org/uniprot/P50516) |  | + |  |  |  |
|  | WD repeat-containing protein 47 | WDR47 | [Q8CGF6](http://www.uniprot.org/uniprot/Q8CGF6) |  | + |  |  |  |
|  |  |  |  |  |  |  |  |  |
|  |  |  |  |  |  |  |  |  |
|  |  |  |  |  |  |  |  |  |
|  |  |  |  |  |  |  |  |  |
