# Supplementary material for: The Progestin Receptor Interactome in the Female Mouse Hypothalamus: Interactions with Synaptic Proteins Are Isoform Specific and Ligand Dependent
Source: eNeuro. 2017 Sep 20;4(5):ENEURO.0272-17.2017. doi: 10.1523/ENEURO.0272-17.2017 (PMC5605756; doi:10.1523/ENEURO.0272-17.2017)
Supplement: Table 3-1 [file enu005172413so4.doc]

| **Table 3-1** |  |  |  |  |
| --- | --- | --- | --- | --- |
| **Protein Name** | **UniProt Symbol** | **UniProt ID** | **PR-A (no ligand/ R5020)** | **PR-B (no ligand/ R5020)** |
| Caspase-3 | CASP3 | P70677 | + | + |
| Caveolin-1^p^ | CAV1^p^ | [P49817](http://www.uniprot.org/uniprot/P49817) | + | + |
| Histone lysine demethylase PHF8 | PHF8 | [Q80TJ7](http://www.uniprot.org/uniprot/Q80TJ7) | + | + |
| Protein S100-A7 | S100A7 | P31151 | + | + |
| Ribonucleoside-diphosphate reductase subunit M2 | RIR2 | P11157 | + | + |
| Serine-protein kinase ATM | ATM | Q62388 | + | + |
| Serine/threonine-protein kinase ATR^p^ | ATR^p^ | [Q9JKK8](http://www.uniprot.org/uniprot/Q9JLN9) | + | + |
| Signal transducer and activator of transcription 5A | STA5A | P42230 | + | + |
| Suppressor of cytokine signaling 1 | SOCS1 | O35716 | + | + |
| β-catenin-1^p^ | CTNB1^p^ | [Q02248](http://www.uniprot.org/uniprot/Q02248) | + | + |
| Integrin-linked protein kinase | ILK | O55222 | + |  |
| Nuclear factor NF-kappa-B p105 subunit | NFKB1 | [P25799](http://www.uniprot.org/uniprot/P25799) | + |  |
| Signal transducer and activator of transcription 3 | STAT3^p^ | [P42227](http://www.uniprot.org/uniprot/P42227) | + |  |
| Vascular endothelial growth factor receptor 2 | VEGFR2 | [P35918](http://www.uniprot.org/uniprot/Q00731) | + |  |
| 5'-AMP-activated protein kinase catalytic subunit α1 | AAPK1^p^ | [Q5EG47](http://www.uniprot.org/uniprot/Q9Z1E4) |  | + |
| 3-oxo-5-alpha-steroid 4-dehydrogenase 1 | S5A1 | [Q99N99](http://www.uniprot.org/uniprot/Q99N99) |  | + |
| 5'-AMP-activated protein kinase subunit β1^p^ | AAKB1 | [Q9R078](http://www.uniprot.org/uniprot/Q9R078) |  | + |
| Aldehyde dehydrogenase | AL1A1 | P24549 |  | + |
| Aldehyde oxidase 1 | AOXA | O54754 |  | + |
| ALK tyrosine kinase receptor^p^ | ALK | [P97793](http://www.uniprot.org/uniprot/P97793) |  | + |
| Annexin A1 | ANXA1 | [P10107](http://www.uniprot.org/uniprot/P10107) |  | + |
| Apoptosis regulator BAX | BAX | [Q07813](http://www.uniprot.org/uniprot/Q07813) |  | + |
| Apoptosis regulator Bcl-2^p^ | BCL2^p^ | P10417 |  | + |
| Aurora kinase C; Aurora kinase B; Aurora kinase A^p^ | AURKA^p^ | [P97477](http://www.uniprot.org/uniprot/P97477) |  | + |
| Bcl2-associated agonist of cell death^p^ | BAD^p^ | Q61337 |  | + |
| Caveolin-1 | CAV1 | [P49817](http://www.uniprot.org/uniprot/P49817) |  | + |
| Cellular tumor antigen p53^p^ | P53^p^ | [P02340](http://www.uniprot.org/uniprot/P02340) |  | + |
| CREB-binding protein | CBP | P45481 |  | + |
| Cyclin-dependent kinase inhibitor 1A | CDN1A | P39689 |  | + |
| Cyclin-dependent kinase inhibitor 1B | CDN1B | P46414 |  | + |
| Dual specificity mitogen-activated protein kinase kinase 1 | MP2K1 | [P31938](http://www.uniprot.org/uniprot/P47811) |  | + |
| Epidermal growth factor receptor | EGFR | Q01279 |  | + |
| Epidermal growth factor receptor^p^ | EGFR^p^ | [Q01279](http://www.uniprot.org/uniprot/Q01279) |  | + |
| Glycine dehydrogenase (decarboxylating), mitochondrial | GCSP | [Q91W43](http://www.uniprot.org/uniprot/Q91W43) |  | + |
| Glycogen synthase kinase-3α | GSK3A | [Q2NL51](http://www.uniprot.org/uniprot/Q2NL51) |  | + |
| Hepatocyte growth factor receptor | MET | [P16056](http://www.uniprot.org/uniprot/P16056) |  | + |
| Histone deacetylase 4 | HDAC4 | [Q6NZM9](http://www.uniprot.org/uniprot/Q923E4) |  | + |
| Integrin α-V | ITAV | [P43406](http://www.uniprot.org/uniprot/P43406) |  | + |
| Mast/stem cell growth factor receptor Kit | KIT | [P05532](http://www.uniprot.org/uniprot/P05532) |  | + |
| Mitogen-activated protein kinase 8^p^ | MK08^p^ | [Q91Y86](http://www.uniprot.org/uniprot/P47811) |  | + |
| Paired amphipathic helix protein Sin3b | SIN3B | Q62141 |  | + |
| Platelet-derived growth factor receptor α | PGFRA | [P26618](http://www.uniprot.org/uniprot/P26618) |  | + |
| Receptor tyrosine-protein kinase erbB-2^p^ | ERBB2^p^ | [P70424](http://www.uniprot.org/uniprot/P70424) |  | + |
| Receptor tyrosine-protein kinase erbB-3^p^ | ERBB3 | Q61526 |  | + |
| Retinoblastoma-associated protein^p^ | RB^p^ | [P13405](http://www.uniprot.org/uniprot/P13405) |  | + |
| Serine-protein kinase ATM^p^ | ATM^p^ | [Q62388](http://www.uniprot.org/uniprot/Q62388) |  | + |
| Set1/Ash2 histone methyltransferase complex subunit ASH2 | ASH2L | [Q91X20](http://www.uniprot.org/uniprot/Q91X20) |  | + |
| Signal transducer and activator of transcription 1-α/β^p^ | STAT1 | P42225 |  | + |
| Trans-acting T-cell-specific transcription factor GATA-3 | GATA3 | [P23772](http://www.uniprot.org/uniprot/P23772) |  | + |
| Tumor necrosis factor ligand superfamily member 11 | TNF11 | [O35235](http://www.uniprot.org/uniprot/P06804) |  | + |
| Tyrosine-protein kinase receptor UFO | UFO | Q00993 |  | + |
